# Global, regional, and national burden of pediatric nasopharyngeal carcinoma (1990–2021) and projections of future disease burden trends

**DOI:** 10.3389/fped.2025.1555091

**Published:** 2025-11-12

**Authors:** Yilong Xu, Huaqiang Dai, Qiuyu Chen, Yanling Xu, Yanyu Xu, Sihai Dai, Mingyan Hong

**Affiliations:** Otorhinolaryngology, Quanzhou Maternity and Children's Hospital, Quanzhou, China

**Keywords:** childhood nasopharyngeal carcinoma, incidence trends, global burden, epidemiological transition, age-standardized incidence rate

## Abstract

**Objective:**

Pediatric nasopharyngeal carcinoma (NPC) is an exceedingly rare and often overlooked disease. However, early detection of this condition is a decisive factor in its prognosis. This study aims to quantify the disease burden and epidemiological trends of pediatric nasopharyngeal carcinoma over a 30-year period (1991–2021) and provide projections for future disease burden.

**Methods:**

Comprehensive data on pediatric NPC from 1990 to 2021 were obtained from the Global Burden of Disease study. This dataset includes information on the incidence of pediatric NPC, disaggregated by gender. The Joinpoint regression model was used to identify turning points in epidemiological trends, while decomposition analysis helped identify the factors driving these trends. To forecast future incidence rates, the Norpred model were applied.

**Results:**

In 1990, the estimated global number of pediatric NPC cases was 1,269 (1,119–1,422), with 764 (663–892) cases in boys and 505 (427–592) cases in girls. The global ASIR of pediatric NPC in 1990 was 0.07 (0.06–0.08), with rates of 0.09 (0.07–0.10) in boys and 0.06 (0.05–0.07) in girls. By 2021, the estimated global number of pediatric NPC cases decreased to 966 (796–1,152), including 576 (444–725) cases in boys and 390 (326–501) cases in girls. The global ASIR of pediatric NPC in 2021 was 0.05 (0.04–0.06), with rates of 0.06 (0.04–0.07) in boys and 0.04 (0.03–0.05) in girls. Joinpoint regression analysis indicated stable epidemiological trends from 1990 to 2021, with a slight decline in both sexes. Based on the Nordpred model, the projected 2046 ASIR for pediatric NPC is 0.09 (male: 0.10; female: 0.80), with 1,169 total cases (boys: 680; girls: 489).

**Conclusion:**

The incidence of pediatric NPC is relatively low overall, with a slight downward trend in its epidemiological trajectory.

## 1 Introduction

Nasopharyngeal carcinoma (NPC) is a malignant tumor originating from the mucosal epithelial cells of the nasopharynx, characterized by distinct geographical and ethnic distribution patterns (1). NPC is a pathological ecosystem with multidimensional spatiotemporal features, whose pathogenesis is extremely complex (2). It is highly prevalent in certain regions of Asia and Africa, while Europe and the Americas are considered low-incidence areas (3). NPC is generally believed to occur predominantly in adults aged 40–60 years, with a second smaller peak reported during adolescence/early adulthood in low- to moderate-incidence countries (4). NPC accounts for 20%–50% of pediatric nasopharyngeal malignancies, second only to rhabdomyosarcoma and non-Hodgkin lymphoma (5). Epstein–Barr virus (EBV) infection is a key etiological factor for NPC (6). Serological studies indicate that 88.9% of non-keratinizing NPC cases exhibit EBV DNA positivity (7). Furthermore, dietary intake of salted fish and preserved foods, genetic susceptibility, and environmental carcinogen exposure collaboratively drive multistep carcinogenic mechanisms of NPC (8).

Compared to adult NPC patients, pediatric NPC exhibits unique clinical characteristics and treatment toxicities (9). While long-term survival rates in children and adolescents with advanced-stage NPC are relatively favorable, 28% of survivors develop severe chronic treatment-related complications (10). TNM stage and radiotherapy were the most significant survival predictors (11). Most pediatric NPC patients develop long-term treatment-related late effects (12). Current strategies to reduce early and late toxicities include radiation dose de-escalation for children demonstrating favorable response to induction chemotherapy (13). Its etiology is closely associated with geographical environment, genetic factors, and EBV infection (14). Regardless of whether in high- or low-incidence regions, the most common pathological type of pediatric NPC is non-keratinizing undifferentiated carcinoma, accounting for over 90% of cases and strongly linked to EBV infection (15). Circulating EBV-DNA is typically positive in pediatric NPC patients (16). Most pediatric NPC patients present with locally advanced disease at diagnosis, making systemic treatment based on radical radiotherapy crucial (12).

A study of 420 long-term survivors revealed that pediatric survivors demonstrated markedly superior quality of life compared to adult survivors (17). Combined chemoradiotherapy significantly improves overall survival and locoregional control rates in children with advanced-stage NPC. Even pediatric cases with distant metastasis should receive curative-intent treatment through systemic chemotherapy with concurrent locoregional radiotherapy (18). The overall treatment outcomes for pediatric NPC are better than those for adults (19). Key prognostic factors include age, initial staging, plasma EBV-DNA load, EBV-related antibody levels, treatment modalities, and post-treatment efficacy evaluation (20). The primary causes of treatment failure are local recurrence and distant metastasis, with bone metastasis being the most common (21). Early detection of pediatric NPC is a decisive factor in patient prognosis (22). Due to the low incidence of pediatric NPC and the challenges in long-term follow-up, epidemiological research on pediatric NPC remains limited. This study aims to fill this gap by analyzing the global, regional, and national disease burden of pediatric NPC from 1990 to 2021, utilizing data from the Global Burden of Disease (GBD) study. By exploring the incidence of pediatric NPC, we aim to comprehensively understand its current disease burden. Additionally, we project future trends in pediatric NPC. Understanding these trends will guide the development of targeted interventions to achieve early diagnosis and improve global pediatric health outcomes.

## 2 Methods

### 2.1 Materials

The 2021 Global Burden of Disease Study offers a comprehensive estimation of the incidence rates of pediatric NPC. These estimates are derived from data collected during the GBD 2021 study, which is accessible through the GBD Results Viewer at https://vizhub.healthdata.org/gbd-results/ (23). The database was most recently accessed on December 26, 2024. The data sources for pediatric NPC encompass a wide array of records from various countries, including hospitalization data, emergency room visits, insurance claims, surveys, and vital registration systems (24).

This study was conducted in accordance with the World Medical Association's Helsinki Declaration. This study employed anonymized data curated by the Institute for Health Metrics and Evaluation (IHME) at the University of Washington. The research protocol, which included a waiver of informed consent, was rigorously reviewed and approved by the Institutional Review Board (IRB) of the University of Washington. The research protocol, which included a waiver of informed consent, was rigorously reviewed and approved by the Institutional Review Board (IRB) of the University of Washington. The GBD 2021 offers an extensive and systematic analysis of 369 diseases and injuries, as well as 87 associated risk factors, meticulously evaluated across 204 countries and territories. The GBD research team has comprehensively documented their methodological framework and publicly disseminated age-standardized incidence rate estimates for these health conditions (25).

Based on the “Cause of death of injury” category in the GBD 2021 database, we extracted the number of incident cases of NPC for three age groups: <5 years, 5–9 years, and 10–14 years. Using population data, we calculated the total number of incident cases and age-standardized incidence rate (ASIR, per 100,000 population) for pediatric NPC (0–14 years). ASIR were calculated to enable comparisons across regions and time periods by accounting for variations in population age structures.

To enable meaningful comparative analysis, the Global Burden of Disease project organizes countries and regions according to their Socio-Demographic Index (SDI). This index divides nations into five distinct SDI tiers: high SDI (>0.81), upper-middle SDI (0.70–0.81), middle SDI (0.61–0.69), lower-middle SDI (0.46–0.60), and low SDI (<0.46). This systematic classification framework not only underscores the shared epidemiological trends but also illuminates the disparities in disease patterns across diverse countries and regions (26).

### 2.2 Statistical analysis

First, the incidence rates of NPC in children across three age groups (<5years, 5–9, and 10–14 years) stratified by gender were extracted from the GBD database. ASIR of pediatric NPC were subsequently calculated using the incidence rates for these three age groups. To illustrate the gender-specific incidence rates across the <5 years, 5–9, and 10–14 age groups, a pyramid chart was utilized. Additionally, a world map was employed to visually represent the global distribution of age-standardized NPC incidence rates by gender for the years 1990 and 2021, highlighting temporal and spatial trends.

Joinpoint regression analysis was conducted using Joinpoint software to identify potential inflection points in the temporal trends of pediatric NPC incidence (27). To elucidate the underlying drivers of these epidemiological shifts, decomposition analysis was systematically applied (28). Furthermore, predictive modeling was performed using the Nordpred model to project the age-standardized incidence rates and the estimated number of pediatric NPC cases over the next three decades, extending to 2046, with stratification by sex (29). These analytical approaches provided a comprehensive framework for understanding and forecasting the evolving burden of pediatric NPC.

All analyses in this study were conducted using R software (R Core Team, version 4.4.2, Vienna, Austria), with a *P*-value of less than 0.05 considered statistically significant.

## 3 Results

**Figure 1:**
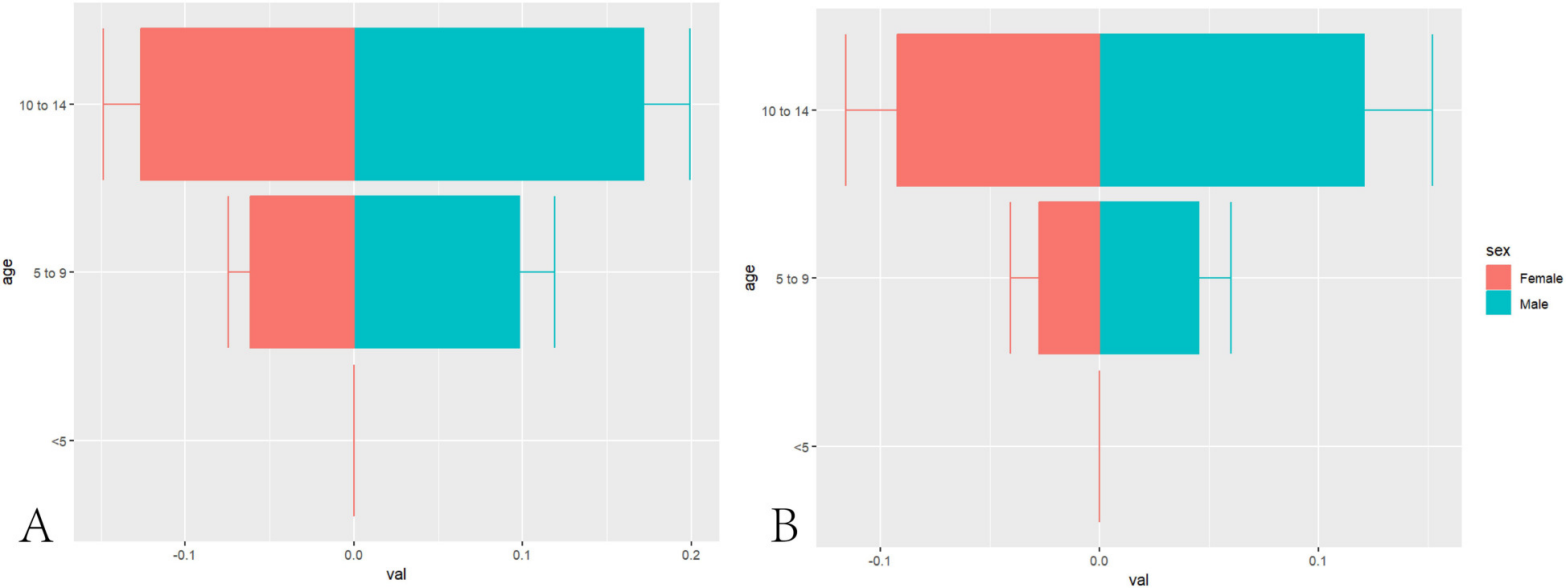
Age and gender distribution of global NPC incidence in children. **(A)** Distribution of Pediatric NPCs in Different Age Groups in 1990. **(B)** Distribution of Pediatric NPCs in Different Age Groups in 2021.

**Figure 2:**
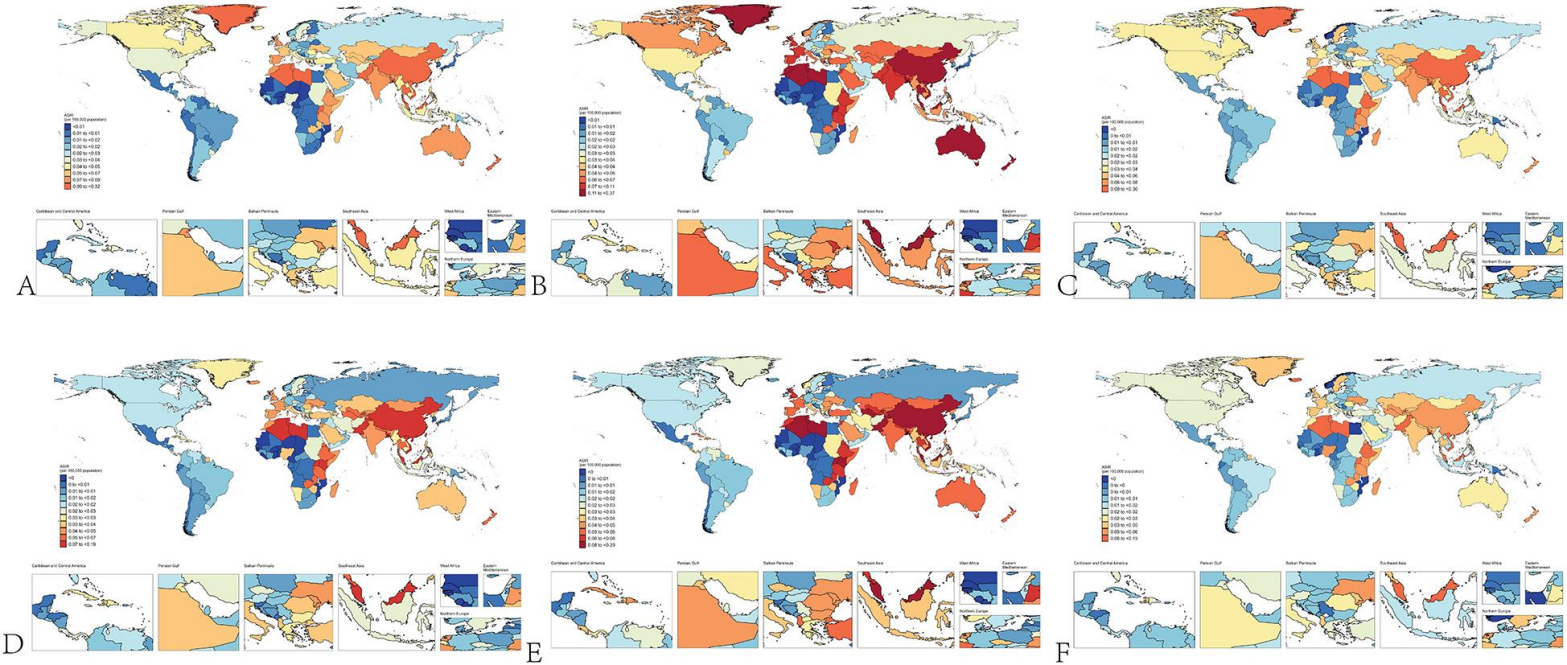
Global distribution of NPC incidence in children. **(A)** Incidence of NPC in all children in 1990. **(B)** Incidence of NPC in male children in 1990. **(C)** Incidence of NPC in female children in 1990. **(D)** Incidence of NPC in all children in 2021. **(E)** Incidence of NPC in male children in 2021. **(F)** Incidence of NPC in female children in 2021.

In 1990, the estimated global number of pediatric NPC cases was 1,269 (1,119–1,422), with 764 (663–892) cases in boys and 505 (427–592) cases in girls. The global ASIR of pediatric NPC in 1990 was 0.07 (0.06–0.08), with rates of 0.09 (0.07–0.10) in boys and 0.06 (0.05–0.07) in girls. By 2021, the estimated global number of pediatric NPC cases decreased to 966 (796–1,152), including 576 (444–725) cases in boys and 390 (326–501) cases in girls. The global ASIR of pediatric NPC in 2021 was 0.05 (0.04–0.06), with rates of 0.06 (0.04–0.07) in boys and 0.04 (0.03–0.05) in girls. Table 1 presents the number of cases and ASIR of pediatric NPC globally and across different SDI regions in 1990 and 2021. Supplementary Table S1 presents the disease burden of pediatric NPCs in 204 countries and regions worldwide in 1990 and 2021. Figure 1 illustrates the incidence rates of pediatric NPC by gender and age group. Figure 2 depicts the global distribution of age-standardized incidence rates of pediatric NPC in 1990 and 2021.

**Table 1:** The ASIR and total incidence number of pediatric NPC in the global and SDI regions in 1990 and 2021.

Global	1990		2021	
	Total incidence (95% CI)	ASIR (95% CI)	Total incidence (95% CI)	ASIR (95% CI)
Both	1,269 (1,119–1,422)	0.07 (0.06–0.08)	966 (796–1,152)	0.05 (0.04–0.06)
Male	764 (663–892)	0.09 (0.07–0.10)	576 (444–725)	0.06 (0.04–0.07)
Female	505 (427–592)	0.06 (0.05–0.07)	390 (326–501)	0.04 (0.03–0.05)
High SDI	1990		2021	
	Total incidence (95% CI)	ASIR (95% CI)	Total incidence (95% CI)	ASIR (95% CI)
Both	78 (73–85)	0.04 (0.04–0.05)	44 (40–49)	0.03 (0.02–0.03)
Male	47 (43–51)	0.05 (0.04–0.05)	26 (23–29)	0.03 (0.03–0.03)
Female	32 (29–35)	0.04 (0.03–0.04)	18 (17–21)	0.02 (0.02–0.02)
High-middle SDI	1990		2021	
	Total incidence (95% CI)	ASIR (95% CI)	Total incidence (95% CI)	ASIR (95% CI)
Both	254 (217–295)	0.09 (0.08–0.11)	143 (112–185)	0.06 (0.05–0.08)
Male	170 (137–208)	0.12 (0.10–0.15)	96 (68–131)	0.08 (0.06–0.11)
Female	84 (69–105)	0.06 (0.05–0.08)	48 (37–63)	0.04 (0.03–0.06)
Middle SDI	1990		2021	
	Total incidence (95% CI)	ASIR (95% CI)	Total incidence (95% CI)	ASIR (95% CI)
Both	501 (444–565)	0.09 (0.08–0.10)	292 (230–357)	0.05 (0.04–0.06)
Male	319 (277–370)	0.11 (0.09–0.12)	190 (136–253)	0.06 (0.05–0.09)
Female	182 (155–213)	0.06 (0.06–0.08)	102 (84–137)	0.04 (0.03–0.05)
Low-middle SDI	1990		2021	
	Total incidence (95% CI)	ASIR (95% CI)	Total incidence (95% CI)	ASIR (95% CI)
Both	299 (226–375)	0.06 (0.05–0.08)	288 (226–361)	0.05 (0.04–0.06)
Male	162 (114–223)	0.07 (0.05–0.09)	162 (120–215)	0.05 (0.04–0.07)
Female	137 (96–188)	0.06 (0.04–0.08)	126 (94–175)	0.04 (0.03–0.06)
Low SDI	1990		2021	
	Total incidence (95% CI)	ASIR (95% CI)	Total incidence (95% CI)	ASIR (95% CI)
Both	136 (94–183)	0.06 (0.04–0.08)	199 (147–262)	0.04 (0.03–0.06)
Male	67 (41–100)	0.06 (0.04–0.09)	103 (71–144)	0.04 (0.03–0.06)
Female	70 (44–98)	0.06 (0.04–0.09)	96 (70–141)	0.04 (0.03–0.06)

**Figure 3:**
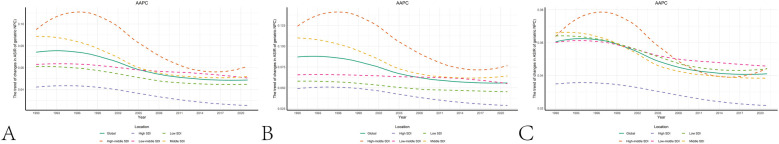
The results of joinpoint regression. **(A)** Changes in the trend of pediatric NPC incidence globally and across different SDI regions. **(B)** Changes in the trend of male pediatric NPC incidence globally and across different SDI regions. **(C)** Changes in the trend of female pediatric NPC incidence globally and across different SDI regions.

Joinpoint regression analysis revealed that the epidemiological trends of pediatric NPC remained relatively stable from 1990 to 2021, with a slight downward trend observed in both boys and girls. The Joinpoint regression results are presented in Figure 3 and Table 2.

**Table 2:** The average annual percent change in the incidence of pediatric NPC globally and across different SDI regions from 1990 to 2021.

Location	Gender	AAPC (95% CI)
Global	Both	−1.34 (−1.39 to −1.29)
	Male	−1.42 (−1.48 to −1.36)
	Female	−1.23 (−1.3 to −1.16)
High SDI	Both	−1.61 (−1.66 to −1.56)
	Male	−1.69 (−1.74 to −1.64)
	Female	−1.53 (−1.59 to −1.43)
High-middle SDI	Both	−1.41 (−1.59 to −1.24)
	Male	−1.49 (−1.69 to −1.3)
	Female	−1.29 (−1.48 to −1.11)
Middle SDI	Both	−1.67 (−1.74 to −1.6)
	Male	−1.67 (−1.73 to −1.61)
	Female	−1.79 (−1.88 to −1.69)
Low-middle SDI	Both	−0.76 (−0.84 to −0.69)
	Male	−0.59 (−0.68 to −0.49)
	Female	−0.96 (−1.06 to −0.87)
Low SDI	Both	−1.04 (−1.16 to −0.93)
	Male	−0.86 (−0.99 to −0.74)
	Female	−1.15 (−1.27 to −1.02)

**Figure 4:**
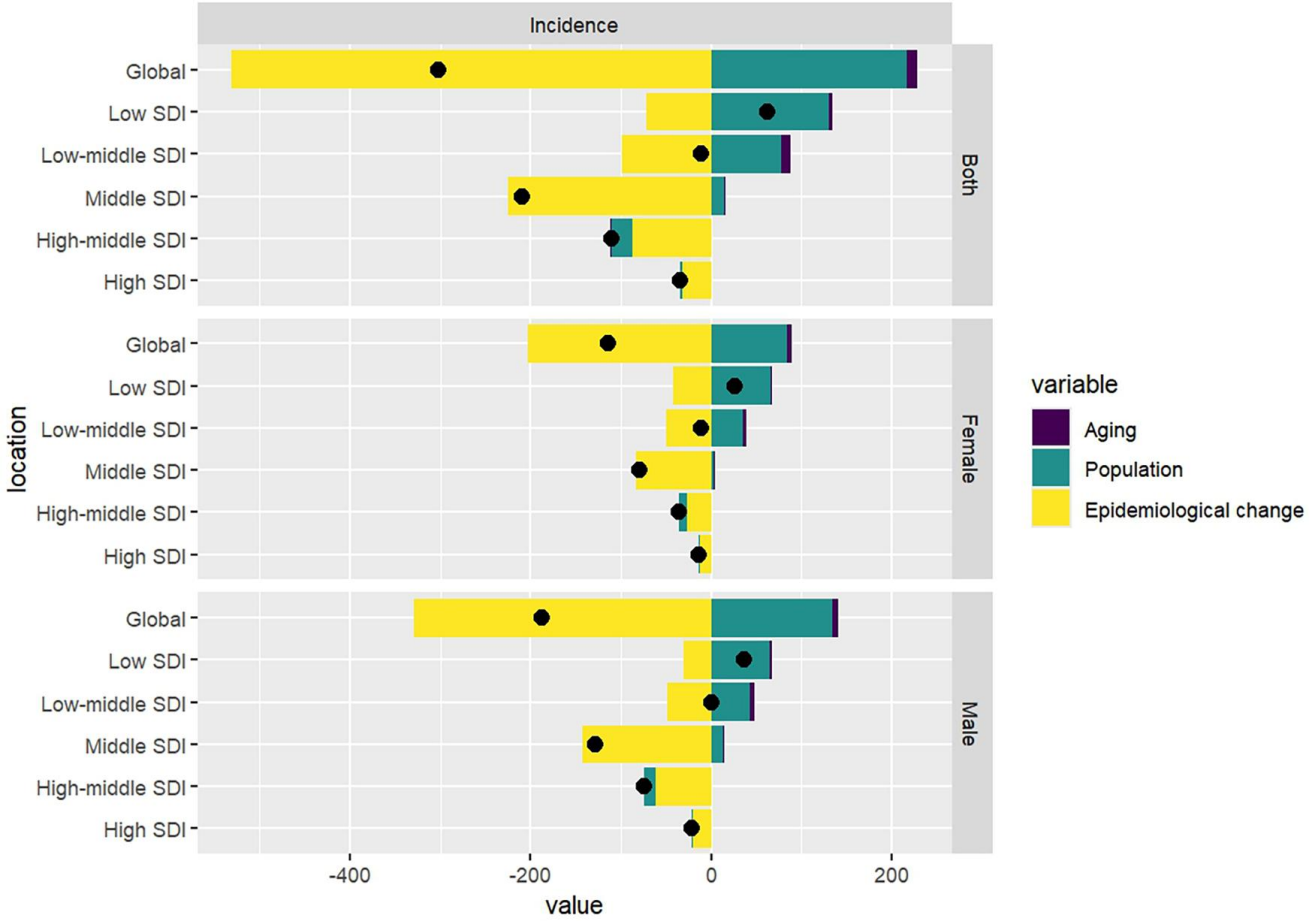
Results of the decomposition analysis.

Decomposition analysis revealed that, while population growth are recognized as driving factors for the increase in the incidence of pediatric NPC globally, the overall epidemiological trend of pediatric NPC has shown a decline. Specifically, the incidence rates of pediatric NPC have decreased globally; however, in low SDI regions, an upward trend in incidence rates has been observed. In these low SDI regions, population growth emerged as the primary driver contributing to the increase in pediatric NPC cases. The results of the decomposition analysis are presented in Table 3 and Figure 4.

**Table 3:** Decomposition analysis results of incidence rate of pediatric NPC in the world and different SDI regions from 1990 to 2021.

Location	Gender	Overall difference	Aging	Population	Epidemiological change
Global	Both	−302.61	11.92 (−3.94%)	216.56 (−71.56%)	−531.09 (175.5%)
	Male	−188.05	6.86 (−3.65%)	133.95 (−71.23%)	−328.86 (174.87%)
	Female	−114.56	5.11 (−4.46%)	83.8 (−73.15%)	−203.47 (177.61%)
High SDI	Both	−34.42	0.52 (−1.52%)	−2.71 (7.88%)	−32.23 (93.65%)
	Male	−21.02	0.32 (−1.5%)	−1.56 (7.4%)	−19.78 (94.1%)
	Female	−13.4	0.21 (−1.56%)	−1.14 (8.53%)	−12.46 (93.03%)
High-middle SDI	Both	−110.74	−0.98 (0.88%)	−22.84 (20.63%)	−86.92 (78.49%)
	Male	−74.47	−0.52 (0.7%)	−12.82 (17.22%)	−61.12 (82.08%)
	Female	−36.28	−0.44 (1.2%)	−8.87 (24.45%)	−26.97 (74.35%)
Middle SDI	Both	−209	2.24 (−1.07%)	14.08 (−6.74%)	−225.32 (107.81%)
	Male	−128.95	1.48 (−1.15%)	12.63 (−9.8%)	−143.06 (110.94%)
	Female	−80.05	0.81 (−1.01%)	2.91 (−3.63%)	−83.76 (104.64%)
Low-middle SDI	Both	−10.85	9.5 (−87.51%)	78.03 (−718.87%)	−98.38 (906.38%)
	Male	0.2	5.17 (2,606.83%)	43.25 (21,786.17%)	−48.22 (−24,293%)
	Female	−11.05	4.33 (−39.21%)	34.82 (−315.02%)	−50.2 (454.23%)
Low SDI	Both	62.44	4.52 (7.24%)	130.31 (208.71%)	−72.4 (−115.95%)
	Male	36.2	2.08 (5.74%)	64.88 (179.24%)	−30.76 (−84.97%)
	Female	26.24	2.46 (9.37%)	65.45 (249.44%)	−41.67 (−158.81%)

**Figure 5:**
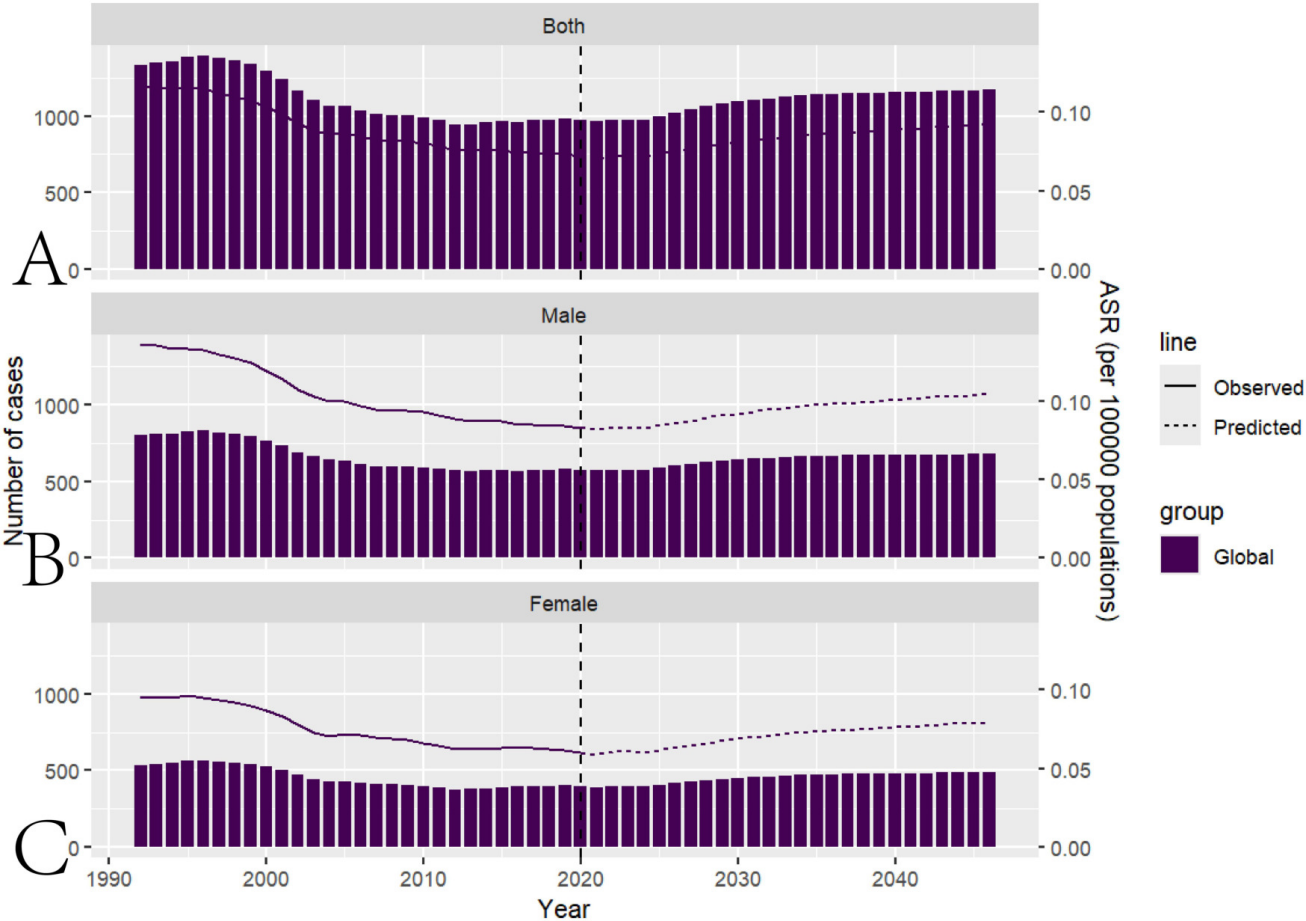
Prediction of future pediatric NPC incidence using the nordpred model. **(A)** Projected global disease burden of pediatric NPC using the Nordpred model. **(B)** Projected global disease burden of male pediatric NPC based on the Nordpred model. **(C)** Projected global disease burden of female pediatric NPC based on the Nordpred model.

Based on the Nordpred model, the predicted ASIR for pediatric NPC by 2046 is 0.09. Among these, the ASIR for male pediatric NPC is 0.10 and for female pediatric NPC, it is 0.80. The total number of pediatric NPC predicted by 2046 is 1169, with 680 cases in boys and 489 cases in girls. The predictions for future pediatric NPC incidence are presented in Figure 5 and Supplementary Table S2.

## 4 Discussion

In our study, we first investigated the relationship between age, gender, and the incidence of NPC in children. We found that the ASIR of NPC was 0 in patients under five years of age. However, as age increased, the incidence of NPC gradually rose, with the incidence rate in patients aged 10–14 years being significantly higher than that in patients aged 5–9 years. EBV, a widely prevalent virus, is closely associated with the development of NPC (30). According to statistics, approximately 90% of NPC patients are positive for EBV antibodies (31). The probability of EBV infection increases with age (32), which may explain why the incidence of NPC is higher in patients aged 10–14 years compared to those aged 5–9 years. It must be recognized, however, that cancer constitutes a complex pathological ecosystem involving ecological and evolutionary dynamics, wherein viral infections such as EBV serve merely as one subset of external environmental triggers (33). Notably, EBV infection does not invariably lead to NPC development in infected individuals. Moreover, EBV infection may not constitute an initiating event, as emerging evidence indicates that chromosomal alterations or other predisposing factors may precede viral infection. Artificial intelligence technologies hold significant potential for early diagnosis of nasopharyngeal carcinoma (34).

Our study also found that, although the incidence rates were generally low, the ASIR of NPC in boys was higher than that in girls. This is consistent with the overall epidemiological trend of NPC in the general population, where the incidence of NPC is higher in males than in females (35). Males may have a higher susceptibility to certain genetic variants associated with NPC (e.g., HLA genes), which could contribute to their higher incidence rates (36). Through the global distribution map of childhood NPC incidence, we observed that childhood NPC is more prevalent in only a few countries, such as Greenland, China, and Algeria, regardless of the year (1990 or 2021) or gender (boys or girls). Therefore, targeted preventive measures for children in these countries may be essential.

Our study also revealed that the global incidence trend of childhood NPC remained relatively stable from 1990 to 2021, with a slight decline. This may be attributed to several factors. First, increased awareness of EBV infection in high SDI regions may have led to reduced EBV infection rates through vaccination and health education, thereby contributing to the decline in NPC incidence (37). In high-incidence regions, such as southern China, the consumption of preserved foods (e.g., salted fish) is strongly associated with NPC development (38). The growing awareness of healthy dietary habits may have reduced the intake of such foods, further lowering NPC incidence. Additionally, environmental improvements in some high-SDI regions may have reduced air pollution and exposure to environmental carcinogens, thereby decreasing NPC risk (39). Lastly, the global decline in fertility rates may have led to a reduction in the proportion of the child population, which could have contributed to a decrease in the absolute number of NPC cases (40).

However, it is important to note that while the ASIR and the number of cases of childhood NPC have shown a declining trend globally, the number of childhood NPC cases in low-SDI regions has increased. Decomposition analysis suggests that population growth is the primary driver of the rising number of cases in low-SDI regions. However, population growth has been a global phenomenon over the past three decades (41). Compared to high-SDI regions, factors such as limited healthcare resources, higher EBV infection rates, and environmental exposures in low-SDI regions may be the underlying reasons for the continued increase in childhood NPC cases in these areas (42). To further prevent NPC, initiatives targeting EBV prevention should be prioritized (43). Additionally, the association between NPC and human papillomavirus (HPV) warrants further investigation. HPV genotypes 16 and 18 are predominant in HPV-positive NPC cases (44). Notably, HPV positivity is more common in non-endemic regions like North America, suggesting distinct etiological pathways compared to EBV-driven NPC in endemic areas (45).

Using the Nordpred model, we estimated that by 2046, the ASIR for pediatric NPC would be 0.09. Specifically, the ASIR for male pediatric NPC is projected to be 0.10, while for female pediatric NPC, it is expected to be 0.08. The total number of pediatric NPC cases predicted by 2046 is 1,169, with 680 cases in boys and 489 cases in girls. To further reduce the incidence of childhood NPC, continued efforts to control air pollution, reduce the consumption of preserved foods, and prevent EBV infection should be considered (1), particularly in low SDI regions.

This study relies on data from the GBD research. While it benefits from high-quality estimates of pediatric NPC, it also faces certain inevitable limitations. Firstly, the GBD research categorizes data units based on countries and regions, lacking information on racial demographics and potentially overlooking the impact of race on the incidence of pediatric NPC. Consequently, analyzing and comparing global trends and changes in the incidence rate of pediatric NPC, as well as examining variations among different SDI levels and age groups within each ethnic group, becomes a challenge. Secondly, early data or data from countries and regions with lower levels of development may be subject to inaccuracies. These limitations should be taken into consideration when interpreting the findings of this study.

## 5 Conclusion

The incidence of pediatric NPC is relatively low overall, with a slight downward trend in its epidemiological trajectory.

## Data Availability

The original contributions presented in the study are publicly available. This data can be found here: To download the data used in these analyses, please visit the Global Health Data Exchange GBD 2019 data-input sources tool at http://ghdx.healthdata.org/gbd-2019/data-input-sources.
